# DNA Methylation Drives Gene Networks Dysregulated in Alzheimer’s Disease

**DOI:** 10.1002/alz.089178

**Published:** 2025-01-03

**Authors:** Erming Wang, Minghui Wang, Gefei Yu, Abigail Thorpe, Vahram Haroutunian, Bin Zhang

**Affiliations:** ^1^ Icahn School of Medicine at Mount Sinai, New York, NY USA; ^2^ Mount Sinai Center for Transformative Disease Modeling, Icahn School of Medicine at Mount Sinai, New York, NY USA; ^3^ Department of Genetics and Genomic Sciences, Icahn School of Medicine at Mount Sinai, New York, NY USA; ^4^ Icahn Genomics Institute, Icahn School of Medicine at Mount Sinai, New York, NY USA; ^5^ Alzheimer’s Disease Research Center, Icahn School of Medicine at Mount Sinai, New York, NY USA; ^6^ Nash Family Department of Neuroscience, Icahn School of Medicine at Mount Sinai, New York, NY USA; ^7^ Department of Psychiatry, Icahn School of Medicine at Mount Sinai, New York, NY USA; ^8^ MIRECC, Peters VA Medical Center, New York, NY USA

## Abstract

**Background:**

Alzheimer’s disease (AD) is a progressive neurodegenerative disease that inflicts the elderly worldwide. Recent studies revealed the association of abnormal methylomic alterations in AD. However, a systematic and comprehensive study is needed to investigate the effects of methylomic changes on the molecular networks underpinning AD, in particular, in brain regions most vulnerable to AD neuropathology.

**Method:**

We profiled genome‐wide methylomic variations in the parahippocampal gyrus across 201 postmortem control, mild cognitive impaired (MCI) and AD brains in the Mount Sinai Brain Bank (MSBB) cohort. We next investigated the influence of methylomic changes on both gene and protein co‐expression networks. We then integrated the methylomic, epigenomic (ATAC‐seq), transcriptomic and proteomic data to infer the regulatory relationships among DNA methylation, chromatin accessibility, transcription and translation. Finally, we validated our key findings using an independent cohort (Religious Orders Study/Memory and Aging Project (ROSMAP)).

**Result:**

We first demonstrated the existence of significant AD‐associated variation in methylomic sites genome‐wide, which were subsequently summarized into distinct differentially methylated regions (DMRs) that are critical in AD. We then quantified the effect of the DMRs on each gene and each protein as well as gene and protein co‐expression networks. We found that DNA methylation had profound influences on not only the AD‐associated gene/protein modules but also their key regulators in the networks. With the integration of the matched multiomics data, we unveiled that DNA methylation likely influenced gene/protein expression via epigenomic peak domains in AD. Further integration of the matched bulk tissue methylation and single nucleus multiomics data revealed cell‐type specific methylomic regulation of transcriptomics in AD.

**Conclusion:**

Our study is the most extensive genome‐wide investigation on the impact of methylation on AD pathology, and the first integrative survey on the complex interactions between methylation, chromatin accessibility, and multiscale transcriptomic and proteomics networks in AD.

